# A Spatial Exploration of Changes in Drug Overdose Mortality in the United States, 2000–2016

**DOI:** 10.5888/pcd16.180405

**Published:** 2019-03-21

**Authors:** Grete E. Wilt, Brian E. Lewis, Erica E. Adams

**Affiliations:** 1 Centers for Disease Control and Prevention/Agency for Toxic Substances and Disease Registry, Office of the Director, Division of Toxicology and Human Health Sciences, Geospatial Research, Analysis, and Services Program (GRASP), Atlanta, Georgia

**Figure:**
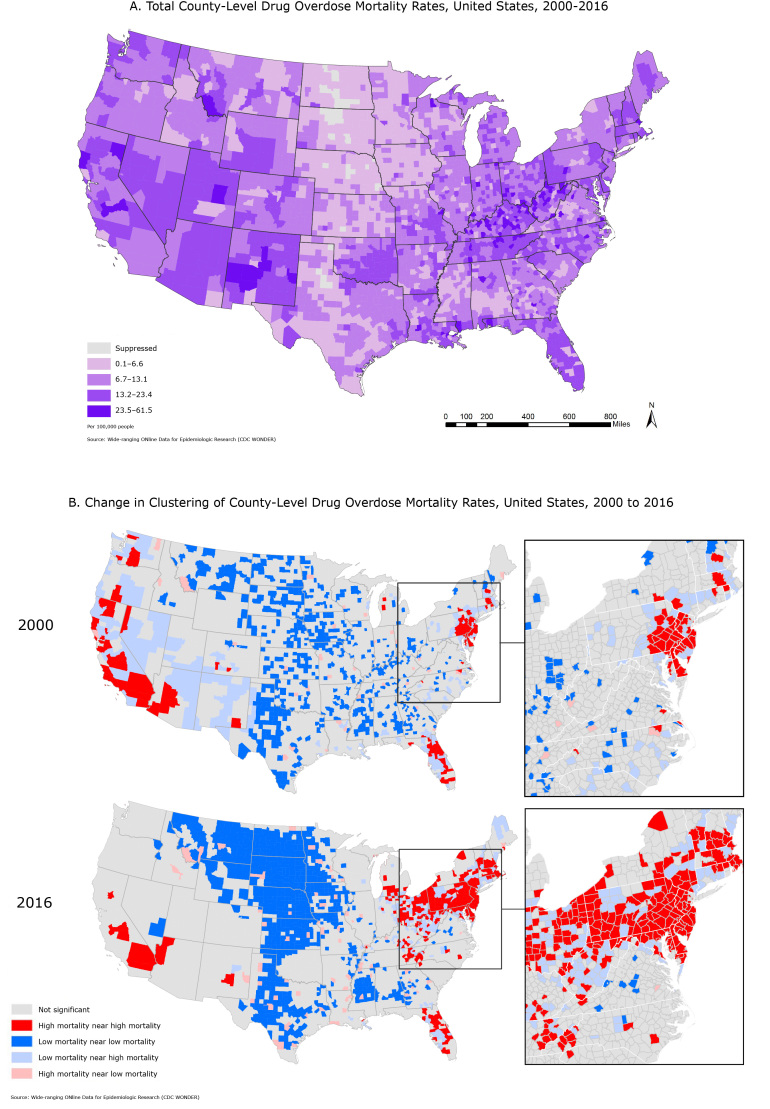
Map A displays all drug overdose mortality in contiguous US counties for 2000–2016. Overall, observed mortality rates were higher in Appalachia and the Southwest. Map B displays clustering in US county-level drug overdose mortality in 2000 adjacent to drug overdose mortality in 2016 to visually compare the geographic change in drug overdose mortality. The magnified images suggest that counties with low mortality rates that are adjacent to those with high mortality rates may be at risk for increased deaths from drug overdose. The results highlight areas that are identified as significant clusters of drug related overdose mortality.

## Background

From 2000 to 2014, rates of drug overdose mortality increased by 137% in the United States. By 2014, 61% of these mortalities were due to opioid overdoses, which have tripled since 2000 (1). Within the last decade, increased amounts of fentanyl in drugs, as well as changes in prescription drug use, have drastically affected the geography of this epidemic. Additionally, the rise in heroin overdoses in recent years has shifted the epidemic into cities (2). The Pew Charitable Trusts reported that in 2015, 38% of all renter households were rent burdened (ie, spending 30% or more of pretax income on rent), a 19% increase since 2001, highlighting the increase of economic immobility in America (3). As overdose mortality rates increased, county-level economic distress worsened (2). Examining this shift in overdose mortalities and their potential drivers through a spatial lens can identify at-risk counties.

We applied geospatial techniques to examine the spatial distribution of drug overdose mortality rates over time to investigate the spread of the epidemic. Use of county-level data promotes policy action, as it allows us to analyze the spread of the epidemic at an intrastate level. We assessed the role of economic distress spatially in correlation to overdose mortality rates. This space–time examination will identify the changing pattern of drug overdose mortality rates in the United States. This analysis aims to inform both national and local policy on allocation of resources for known interventions and to prepare communities where we anticipate high mortality rates.

## Methods

We used age-adjusted all-drug overdose mortality data at the county level. County-scale data provide more detail than state-scale data. Data came from Wide-ranging ONline Data for Epidemiologic Research (CDC WONDER). CDC WONDER is a public resource developed for research, decision making, and program evaluation (wonder.cdc.gov). To encompass all drug–related deaths, our database comprised *International Classification of Diseases, 10th revision* (ICD-10) underlying cause-of-death codes for all-drug overdose: X40–44 (unintentional), X60–64 (suicide), X85 (homicide), or Y10–Y14 (undetermined intent) based on the Substance Abuse and Mental Health Services Administration and CDC WONDER (4,5). We obtained American Community Survey data on the percentage of renter-occupied housing units whose gross rent as a percentage of household income was greater than or equal to 50% (https://factfinder.census.gov/faces/nav/jsf/pages/download_center.xhtml). This variable, referred to as rent burden, was selected as a proxy for economic distress (4,6). We examined spatial trends in age-adjusted overdose mortality rates by using univariate, bivariate, and differential Moran’s *I* at both the global and local levels. Using the software GeoDa 1.12 and R 3.2, these methods test for spatial autocorrelation, a measure of clustering of a variable with itself or other variables in space (univariate and bivariate) and/or time (differential). Global analyses determine if significant clustering is occurring across the entire study area, and local analyses identify specific locations that clustering is occurring. Moran’s *I* values with a *P* < .05 were deemed significant.

## Main Findings

Local Moran’s *I* tests indicated that both high mortality clusters and high–low clusters (areas of high mortality around areas of low mortality) increased from 2000 to 2016 (Table). Univariate Moran’s *I* showed a moderately strong (*I* approximately > 0.1) significant spatial clustering pattern of 2000, 2010, 2014 and 2016 age-adjusted mortality rates (*P* < .05 for all tests). Bivariate Moran’s *I* using mortality rates and rent burden data also showed significant clustering, which nearly doubled in magnitude from 2010 to 2016 (Table). Differential Moran’s *I* highlighted a growth of significant clustering from 2000 to 2016.

**Table:** Results of Global and Local Moran’s *I* Analysis for Overdose Mortality Rates, United States, 2000–2016

Variable	Univariate^a^				Bivariate^b^			Differential^c^
	2000	2010	2014	2016	2010 and Burden	2014 and Rent Burden	2016 and Burden	2000 and 2016
Moran’s *I*	0.114^d^	0.098^d^	0.133^d^	0.231^d^	0.081^d^	0.112^d^	0.145^d^	0.114^d^
No. of high–high clusters	102	132	200	265	230	254	160	104
No. of low–low clusters	547	505	549	693	487	500	441	174
No. of high–low clusters	39	55	65	72	64	67	67	43
No. of low–high clusters	220	177	200	164	228	197	169	218

a Univariate Moran’s *I* identifies significant outlier clusters of areas with high age-adjusted drug overdose mortality rates surrounded by other areas of high age-adjusted drug overdose mortality rates.

b Bivariate Moran’s *I* identifies significant outlier clusters of areas with high age-adjusted drug overdose mortality rates surrounded by other areas of high percentages of populations who contribute greater than 50% of their income to rent.

c Differential Moran’s *I* identifies significant outlier clusters of areas with high 2016 age-adjusted drug overdose mortality rates surrounded by other areas of high age-adjusted drug overdose mortality rates in 2000.

d *P* < .05.

The differential Moran’s *I* analysis illustrates the pervasive spread of drug overdose mortality over time. The growth of high mortality rate clusters over time occurred in areas that had previous low mortality rates in close proximity to areas with high mortality rates. These low–high clusters continued to grow, which indicates a strong spatial component of drug overdoses and predicts expansion of drug-overdose mortality rates. The increase in nonsignificant areas over time is due to increased mortality rates, leading to reduced outlier detection. Counties with originally low mortality clustering in the West had mortality growth in recent years. Many counties that had significantly low mortality rates in 2000 did not have significantly low rates in 2016. The overall increase in mortality rates decreased the amount of significantly high mortality county clusters. For example, the average overdose-related death rate per 100,000 people increased from 3.6 in 2000 to 12.1 in 2016 in California, 2.6 to 7.7 in Arizona, and 0.85 to 5.8 across the United States, showing that what was once considered a high drug-overdose mortality rate is now likely considered average or even low.

## Action

These maps highlight the overall pattern (Map A) and geographic shift in the drug overdose epidemic, highlighting regions with increases in high mortality rates over the past decade (Map B). Clusters of high mortality rates are expanding into neighboring areas of low mortality rates over time and areas of high rent burden (2). Results of this analysis indicate a changing landscape in the drug overdose epidemic over space and time. This finding is not evident when looking at overall age-adjusted rates for the period. By spatially identifying areas of potential risk, our findings suggest the use of geographic information systems (GIS) to identify locations for preventive interventions and targeting resources at the county level. Lawmakers should target highly rent-burdened neighborhoods in counties with significantly high mortality. We suggest implementing known effective interventions in these counties. Interventions include increased first-responder access to naloxone, establishing supervised injection facilities and substance abuse treatment facilities including Medication-Assisted Treatment (MAT), and increased support for state-run prescription drug monitoring programs. GIS can be used to understand trends in location-based prescription rates and health outcomes, to understand infrastructural and social barriers to treatment access, and to prioritize resources for geographically targeted policy, education, and treatment interventions. Because of the rapid increase of overdose rates in the last 2 decades and limited resources, we must target prevention strategies to counties where spatial analyses have forecasted future high overdose mortality rates.
